# Supplementary material for: Metformin Modifies the Gut Microbiota of Mice Infected with Helicobacter pylori
Source: Pharmaceuticals (Basel). 2021 Apr 3;14(4):329. doi: 10.3390/ph14040329 (PMC8065676; doi:10.3390/ph14040329)
Supplement: Supplementary file 1 [file pharmaceuticals-14-00329-s001.pdf]

Supplementary Materials:

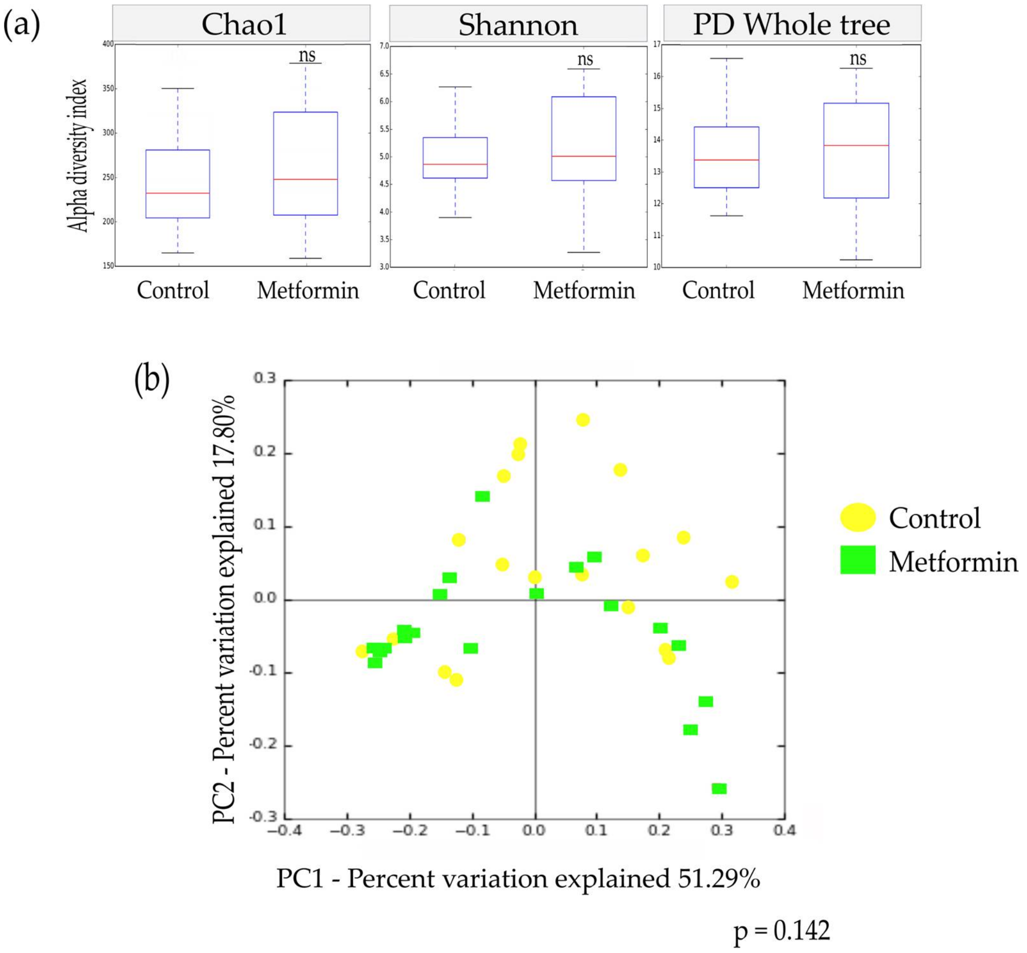

Figure S1. Alpha and beta diversity comparison of fecal microbiota between the metformin and control groups before the beginning of treatment. (**a**) Alpha diversity analyses. Ns, non-significant. Student's *t*-test. (**b**) Principal coordinate analysis (PCoA) plots created using weighted UniFrac distances. Green and yellow dots indicate metformin and control samples, respectively.

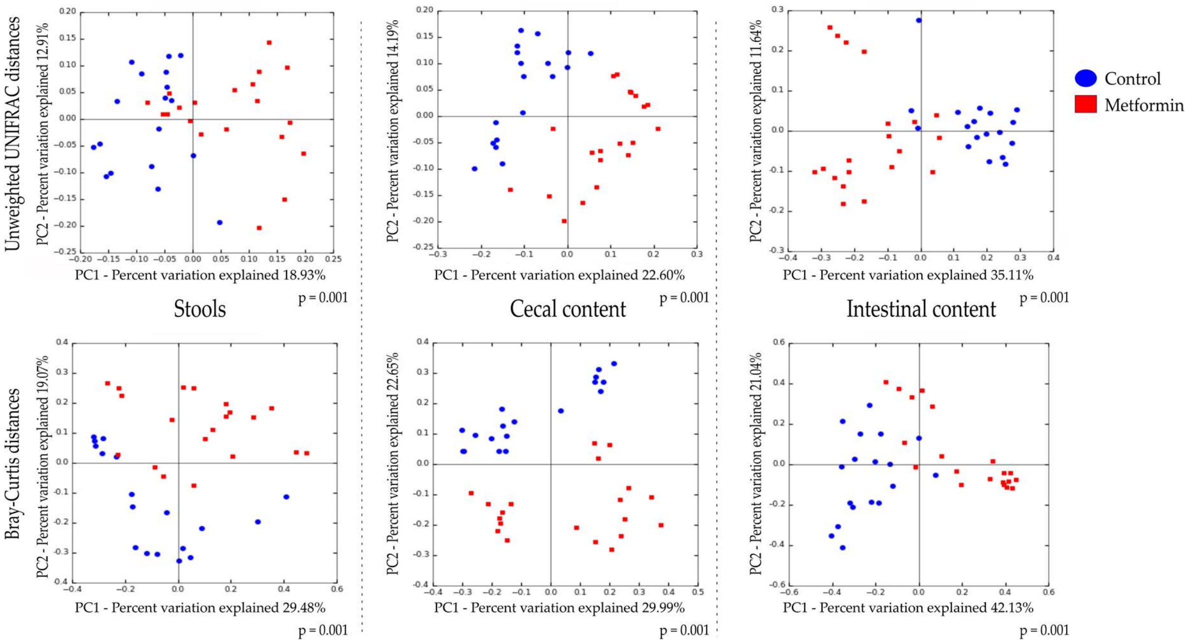

Figure S2. 2D PCoA plots created using unweighted UniFrac and Bray–Curtis distances. Red and blue dots indicate metformin and control samples, respectively. Adonis statistical tests showed significant differences between the two groups (999 permutations; for unweighted Unifrac distances R2 = 0.138, 0.174 and 0.260 for stool, cecal and intestinal content respectively, for Bray-Curtis distances R2 = 0.162, 0.200 and 0.297 for stool, cecal and intestinal content respectively, *p* = 0.001).

Table S1. Comparison of the relative abundance of bacteria between the metformin and control treatment groups at species level for different digestive sites. The comparison was performed with Multiple t test, NS: non-significant (*p* > 0.05).

	Stools				Caecal content				Intestinal content			
		**Control (%)**	**Metformin (%)**	**p**		**Control (%)**	**Metformin (%)**	**p**		**Control (%)**	**Metformin (%)**	**p**
	Bifidobacterium pseudolongum	7.28	28.14	2.83E-04	*Corynebacterium stationis*	4.4E-04	0	NS	*Corynebacterium stationis*	2.38E-03	1.64E-03	NS
	Alistipes indistinctus	0	2.77E-03	NS	*Bifidobacterium pseudolongum*	1.92	18.31	9.00E-06	*Bifidobacterium pseudolongum*	20.79	67.44	<1.00E-06
	Jeotgalicoccus psychrophilus	0.148	0.020	6.12E-03	*Jeotgalicoccus psychrophilus*	0.025	9.13E-03	NS	*Bacillus cereus*	0	1.69E-03	NS
	Staphylococcus aureus	0.026	4.28E603	1.31E-02	*Staphylococcus aureus*	3.09E-03	2.20E-03	NS	*Jeotgalicoccus psychrophilus*	0.207	0.042	NS
	Staphylococcus sciuri	4.16E-03	2.19E-03	NS	*Staphylococcus sciuri*	0	6.21E-04	NS	*Staphylococcus aureus*	0.041	8.55E-03	1.75E-02
	Lactobacillus reuteri	1.89E-03	0	NS	*Defluvitalea saccharophila*	0.058	0.023	8.62E-03	*Staphylococcus sciuri*	5.98E-03	3.73E-03	NS
	Defluvitalea saccharophila	0.165	0.030	8.45E-04	*Ruminococcus gnavus*	2.54	2.55	NS	*Lactobacillus reuteri*	0.020	3.38E-03	1.24E-02
	Ruminococcus gnavus	2.42	2.15	NS	*Butyricicoccus pullicaecorum*	1.61	0.83	4.65E-04	*Streptococcus infantis*	2.04E-03	0	NS
**Species**	*Butyricicoccus pullicaecorum*	0.919	0.745	NS	*Clostridium methylpentosum*	0.013	4.23E-03	3.27E-02	*Defluvitalea saccharophila*	0.11	3.85E-03	1.80E-03
	Clostridium methylpentosum	5.40E-03	0.012	NS	*Enterobacter hormaechei*	0	7.56E-03	NS	*Ruminococcus gnavus*	4.9	0.627	8.58E-04
	Oxalobacter formigenes	4.43E-03	0	2.83E-02	*Escherichia coli*	8.80E-04	8.9E-03	NS	*Butyricicoccus pullicaecorum*	0.22	0.031	5.30E-05
	Enterobacter hormaechei	0	8.95E-03	NS	*Akkermansia muciniphila*	0.048	0.21	2.00E-06	*Helicobacter pylori*	0	3.78E-03	NS
	Escherichia coli	9.11E-03	0.018	NS					*Enterobacter hormaechei*	0	9.61E-03	NS
	Akkermansia muciniphila	0.146	0.248	NS					*Escherichia coli*	0.085	0.084	NS
									Acinetobacter johnsonii	0	9.34E-03	NS
									Pseudomonas veronii	1.40E-03	0	NS
									Akkermansia muciniphila	0.26	0.18	NS

Table S2. Bacterial taxa with LDA scores > 2 in at least two of the three digestives sites in the metformin and control groups. S, stool; CC, cecum; IC, intestine.

Bacterial taxa	Digestive site	LDA score	Group	p
k__Bacteria.p__Actinobacteria.c__Actinobacteria.o__Bifidobacteriales.f__Bifidobacteriaceae.g__Bifidobacterium	IC	4.66	Metformin	7.71E-06
k__Bacteria.p__Actinobacteria.c__Actinobacteria.o__Bifidobacteriales.f__Bifidobacteriaceae.g__Bifidobacterium	S	4.32	Metformin	1.62E-04
k__Bacteria.p__Actinobacteria.c__Actinobacteria.o__Bifidobacteriales.f__Bifidobacteriaceae.g__Bifidobacterium	CC	4.22	Metformin	5.76E-07
k__Bacteria.p__Firmicutes.c__Clostridia.o__Clostridiales.f__Ruminococcaceae.g__Anaerotruncus	S	3.01	Metformin	5.01E-03
k__Bacteria.p__Firmicutes.c__Clostridia.o__Clostridiales.f__Ruminococcaceae.g__Anaerotruncus	CC	3.07	Metformin	8.60E-04
k__Bacteria.p__Verrucomicrobia.c__Verrucomicrobiae.o__Verrucomicrobiales.f__Verrucomicrobiaceae.g__Akkermansia	S	2.09	Metformin	1.09E-02
k__Bacteria.p__Verrucomicrobia.c__Verrucomicrobiae.o__Verrucomicrobiales.f__Verrucomicrobiaceae.g__Akkermansia	CC	2.32	Metformin	2.47E-05
k__Bacteria.p__Actinobacteria.c__Coriobacteriia.o__Coriobacteriales.f__Coriobacteriaceae.g__Adlercreutzia	CC	2.47	Control	5.03E-04
k__Bacteria.p__Actinobacteria.c__Coriobacteriia.o__Coriobacteriales.f__Coriobacteriaceae.g__Adlercreutzia	IC	3.83	Control	2.67E-07
k__Bacteria.p__Actinobacteria.c__Coriobacteriia.o__Coriobacteriales.f__Coriobacteriaceae.g__Adlercreutzia	S	5.6	Control	5.10E-06
k__Bacteria.p__Bacteroidetes.c__Bacteroidia.o__Bacteroidales.f__Rikenellaceae	IC	2.74	Control	2.53E-04
k__Bacteria.p__Bacteroidetes.c__Bacteroidia.o__Bacteroidales.f__Rikenellaceae	S	4.05	Control	4.83E-05
k__Bacteria.p__Firmicutes.c__Bacilli.o__Bacillales.f__Planococcaceae.g__Sporosarcina	CC	2.42	Control	1.61E-04
k__Bacteria.p__Firmicutes.c__Bacilli.o__Bacillales.f__Planococcaceae.g__Sporosarcina	IC	3.37	Control	2.22E-08
k__Bacteria.p__Firmicutes.c__Bacilli.o__Bacillales.f__Planococcaceae.g__Sporosarcina	S	2.49	Control	5.94E-05
k__Bacteria.p__Firmicutes.c__Bacilli.o__Lactobacillales.f__Aerococcaceae.g__Aerococcus	IC	3.37	Control	7.09E-03
k__Bacteria.p__Firmicutes.c__Bacilli.o__Lactobacillales.f__Aerococcaceae.g__Aerococcus	S	3.2	Control	2.83E-03
k__Bacteria.p__Firmicutes.c__Bacilli.o__Lactobacillales.f__Lactobacillaceae.g__Lactobacillus	IC	2.81	Control	4.18E-05
k__Bacteria.p__Firmicutes.c__Bacilli.o__Lactobacillales.f__Lactobacillaceae.g__Lactobacillus	S	2.32	Control	2.80E-03
k__Bacteria.p__Firmicutes.c__Clostridia.o__Clostridiales.f___Mogibacteriaceae	IC	2.84	Control	5.76E-06
k__Bacteria.p__Firmicutes.c__Clostridia.o__Clostridiales.f___Mogibacteriaceae	S	2.34	Control	3.80E-03
k__Bacteria.p__Firmicutes.c__Clostridia.o__Clostridiales.f__Eubacteriaceae.g__Anaerofustis	CC	2.87	Control	4.09E-05
k__Bacteria.p__Firmicutes.c__Clostridia.o__Clostridiales.f__Eubacteriaceae.g__Anaerofustis	IC	3.18	Control	4.82E-06
k__Bacteria.p__Firmicutes.c__Clostridia.o__Clostridiales.f__Eubacteriaceae.g__Anaerofustis	S	2.64	Control	1.09E-07
k__Bacteria.p__Firmicutes.c__Clostridia.o__Clostridiales.f__Lachnospiraceae.g__Defluviitalea	CC	2.76	Control	6.04E-03
k__Bacteria.p__Firmicutes.c__Clostridia.o__Clostridiales.f__Lachnospiraceae.g__Defluviitalea	IC	3,00	Control	5.15E-05
k__Bacteria.p__Firmicutes.c__Clostridia.o__Clostridiales.f__Lachnospiraceae.g__Defluviitalea	S	2.38	Control	1.30E-03
k__Bacteria.p__Firmicutes.c__Clostridia.o__Clostridiales.f__Lachnospiraceae.g__Dorea	CC	2.47	Control	5.48E-03
k__Bacteria.p__Firmicutes.c__Clostridia.o__Clostridiales.f__Lachnospiraceae.g__Dorea	IC	3.23	Control	1.39E-06
k__Bacteria.p__Firmicutes.c__Clostridia.o__Clostridiales.f__Lachnospiraceae.g__Dorea	S	2.62	Control	5.00E-03
k__Bacteria.p__Firmicutes.c__Clostridia.o__Clostridiales.f__Lachnospiraceae.Other	CC	2.81	Control	6.25E-04
k__Bacteria.p__Firmicutes.c__Clostridia.o__Clostridiales.f__Lachnospiraceae.Other	IC	2.92	Control	5.63E-04
k__Bacteria.p__Firmicutes.c__Clostridia.o__Clostridiales.f__Peptococcaceae	CC	2.6	Control	4.30E-05
k__Bacteria.p__Firmicutes.c__Clostridia.o__Clostridiales.f__Peptococcaceae	S	2.65	Control	1.14E-03
k__Bacteria.p__Firmicutes.c__Clostridia.o__Clostridiales.f__Ruminococcaceae.g__Butyricicoccus	CC	2.92	Control	2.86E-03
k__Bacteria.p__Firmicutes.c__Clostridia.o__Clostridiales.f__Ruminococcaceae.g__Butyricicoccus	IC	3.24	Control	4.30E-05

Table S3. OTUs number of bacterial taxa with LDA scores > 2 in at least two of the three digestives sites in the metformin and control groups.

k__Bacteria.p__Actinobacteria.c__Actinobacteria.o__Bifidobacteri ales.f__Bifidobacteriaceae.g__Bifidobacterium			
denovo61	denovo23473	denovo55509	denovo80520
denovo2048	denovo24599	denovo58023	denovo81936
denovo2977	denovo24764	denovo58100	
denovo7075	denovo27348	denovo58147	
denovo7667	denovo27503	denovo59318	
denovo11481	denovo29030	denovo59364	
denovo12076	denovo30094	denovo59616	
denovo12525	denovo30693	denovo63191	
denovo13026	denovo33065	denovo64599	
denovo13373	denovo35631	denovo68732	
denovo13409	denovo36641	denovo69663	
denovo13916	denovo37376	denovo69807	
denovo14154	denovo38295	denovo72552	
denovo18644	denovo38806	denovo73720	
denovo19115	denovo44853	denovo74210	
denovo19865	denovo44862	denovo75425	
denovo20767	denovo47006	denovo76618	
denovo21149	denovo48387	denovo78231	
denovo22125	denovo52447	denovo79144	
denovo23296	denovo54642	denovo79821	

k__Bacteria.p__Firmicutes.c__Cl ostridia.o__Clostridiales.f__Rum inococcaceae.g__Anaerotruncus
denovo5145
denovo13775
denovo26114
denovo30777
denovo32531
denovo39158
denovo44434
denovo56971
denovo63363
denovo67959

k__Bacteria.p__Verrucomicrobia.c__Verrucomicrobiae.o__Verrucom icrobiales.f__Verrucomicrobiaceae.g__Akkermansia
denovo3467

k__Bacteria.p__Actinobacteria.c__Coriobacteriia.o__Coriobacteri ales.f__Coriobacteriaceae.g__Ad lercreutzia
denovo5129
denovo9913
denovo29300
denovo30712
denovo49398
denovo61935
denovo63577
denovo69517
denovo75060
denovo78141

k__Bacteria.p__Bacteroidetes.c__Bacteroidia.o__Bacteroidales.f__Rikenellaceae			
denovo196	denovo26021	denovo53091	denovo73032
denovo3230	denovo28060	denovo54361	denovo73415
denovo3474	denovo28634	denovo55241	denovo74873
denovo3951	denovo29010	denovo56984	denovo76850
denovo4864	denovo30959	denovo57184	denovo76907
denovo4934	denovo31963	denovo57549	denovo78186
denovo6201	denovo32719	denovo58057	denovo80386
denovo7526	denovo33881	denovo59067	
denovo7753	denovo34600	denovo59156	
denovo12707	denovo37333	denovo59402	
denovo14165	denovo37777	denovo60759	
denovo15925	denovo38196	denovo61312	
denovo16810	denovo38368	denovo62648	
denovo17871	denovo40886	denovo63346	
denovo19911	denovo41825	denovo64795	
denovo21512	denovo43124	denovo66070	
denovo22753	denovo44722	denovo66304	
denovo23382	denovo44737	denovo69022	
denovo24486	denovo49221	denovo70600	
denovo25232	denovo50282	denovo72548	

k__Bacteria.p__Firmicutes.c__Ba cilli.o__Bacillales.f__Planococcac eae.g__Sporosarcina
denovo68541

k__Bacteria.p__Firmicutes.c__Ba cilli.o__Lactobacillales.f__Aeroc occaceae.g__Aerococcus
denovo37199
denovo54067

k__Bacteria.p__Firmicutes.c__Ba cilli.o__Lactobacillales.f__Lacto bacillaceae.g__Lactobacillus
denovo9258
denovo24749
denovo32503
denovo47113
denovo48225

k__Bacteria.p__Firmicutes.c__Cl ostridia.o__Clostridiales.f__Mo gibacteriaceae
denovo4410
denovo21302
denovo22624
denovo23338
denovo32927
denovo38797
denovo41671
denovo49299
denovo66327

k__Bacteria.p__Firmicutes.c__Cl ostridia.o__Clostridiales.f__Euba cteriaceae.g__Anaerofustis
denovo41203

k__Bacteria.p__Firmicutes.c__Cl ostridia.o__Clostridiales.f__Lach nospiraceae.g__Defluviitalea
denovo31182

k__Bacteria.p__Firmicutes.c__Cl ostridia.o__Clostridiales.f__Lach nospiraceae.g__Dorea
denovo13194
denovo17940
denovo34843
denovo42510
denovo48083
denovo49645
denovo49898
denovo52940
denovo57746
denovo62725
denovo68402
denovo68960
denovo72958
denovo76059
denovo76336
denovo81423
denovo82146
denovo9175
denovo50374
denovo64883

k__Bacteria.p__Firmicutes.c__Clostridia.o__Clostridiales.f__Lachnospiraceae.Other																
denovo1969	denovo56594	denovo3431	denovo12242	denovo18452	denovo24688	denovo32119	denovo37954	denovo44823	denovo50908	denovo58801	denovo65978	denovo73235	denovo79057	denovo48884	denovo23757	denovo73749
denovo2269	denovo57354	denovo3462	denovo12263	denovo18608	denovo24727	denovo32514	denovo38040	denovo45021	denovo50984	denovo58873	denovo66098	denovo73245	denovo79073	denovo52639	denovo25826	denovo73934
denovo3900	denovo57969	denovo3537	denovo12371	denovo18725	denovo24733	denovo32521	denovo38081	denovo45046	denovo51149	denovo59030	denovo66229	denovo73283	denovo79156	denovo54573	denovo26211	denovo75318
denovo4122	denovo58458	denovo3546	denovo12445	denovo18793	denovo25053	denovo32637	denovo38163	denovo45154	denovo51187	denovo59052	denovo66370	denovo73376	denovo79358	denovo54662	denovo26245	denovo75871
denovo5363	denovo59179	denovo3872	denovo12530	denovo18908	denovo25126	denovo32653	denovo38210	denovo45390	denovo51293	denovo59165	denovo66443	denovo73781	denovo79467	denovo54692	denovo26593	denovo75940
denovo5463	denovo59533	denovo4131	denovo12580	denovo18959	denovo25187	denovo32729	denovo38456	denovo45447	denovo51372	denovo59412	denovo66615	denovo73945	denovo79742	denovo56924	denovo28099	denovo76030
denovo5876	denovo60127	denovo4223	denovo12599	denovo19079	denovo25455	denovo32870	denovo38473	denovo45464	denovo51490	denovo59451	denovo66640	denovo73977	denovo79767	denovo60354	denovo30991	denovo77060
denovo6711	denovo62176	denovo4311	denovo12636	denovo19123	denovo25732	denovo32984	denovo38672	denovo45758	denovo51555	denovo59519	denovo66875	denovo74196	denovo79989	denovo60843	denovo31178	denovo77577
denovo8872	denovo64006	denovo4598	denovo12694	denovo19261	denovo25795	denovo32995	denovo38678	denovo45851	denovo51556	denovo59621	denovo66943	denovo74322	denovo80057	denovo61206	denovo31611	denovo80883
denovo9802	denovo68089	denovo4723	denovo13132	denovo19664	denovo25991	denovo33000	denovo38770	denovo45939	denovo51617	denovo60324	denovo67130	denovo74552	denovo80110	denovo63670	denovo34092	denovo81358
denovo9966	denovo69613	denovo4842	denovo13299	denovo19837	denovo26003	denovo33081	denovo38812	denovo46034	denovo51667	denovo60511	denovo67390	denovo74674	denovo80550	denovo65780	denovo34455	denovo82260
denovo11272	denovo71685	denovo4889	denovo13331	denovo19882	denovo26010	denovo33095	denovo38814	denovo46144	denovo51770	denovo60645	denovo67394	denovo74918	denovo80707	denovo67789	denovo35519	
denovo13892	denovo72458	denovo5557	denovo13541	denovo20160	denovo26220	denovo33148	denovo38827	denovo46203	denovo51813	denovo60647	denovo67647	denovo74943	denovo81261	denovo68755	denovo35762	
denovo15759	denovo72836	denovo5791	denovo13600	denovo20197	denovo26239	denovo33243	denovo38938	denovo46235	denovo51863	denovo60847	denovo67654	denovo75081	denovo81472	denovo69076	denovo36046	
denovo16683	denovo73581	denovo6358	denovo13633	denovo20223	denovo26407	denovo33288	denovo39589	denovo46285	denovo51874	denovo60954	denovo67682	denovo75389	denovo81484	denovo69212	denovo36389	
denovo19078	denovo74433	denovo6374	denovo13673	denovo20404	denovo26506	denovo33645	denovo39657	denovo46298	denovo52076	denovo61078	denovo67748	denovo75410	denovo81532	denovo72110	denovo36555	
denovo21278	denovo75622	denovo6732	denovo13800	denovo20415	denovo26627	denovo33722	denovo39671	denovo46323	denovo52245	denovo61280	denovo67811	denovo75431	denovo81562	denovo73978	denovo36718	
denovo22930	denovo79199	denovo6738	denovo13810	denovo20516	denovo26675	denovo33790	denovo39960	denovo46712	denovo52638	denovo61373	denovo67885	denovo75590	denovo81858	denovo75129	denovo37217	
denovo23922	denovo79328	denovo6741	denovo13859	denovo20548	denovo26754	denovo33831	denovo40149	denovo46753	denovo52670	denovo61449	denovo68138	denovo75753	denovo81949	denovo75245	denovo38610	
denovo24519	denovo79655	denovo6771	denovo13884	denovo20777	denovo26762	denovo34005	denovo40154	denovo46909	denovo52799	denovo61706	denovo68436	denovo75948	denovo665	denovo76813	denovo38621	
denovo24828	denovo80164	denovo7083	denovo13896	denovo21155	denovo26796	denovo34038	denovo40201	denovo46976	denovo52929	denovo61847	denovo69233	denovo75971	denovo1702	denovo77543	denovo38823	
denovo25523	denovo81109	denovo7119	denovo14027	denovo21215	denovo26869	denovo34151	denovo40417	denovo47094	denovo53117	denovo61952	denovo69265	denovo76091	denovo7789	denovo77706	denovo39896	
denovo26974	denovo81828	denovo7269	denovo14115	denovo21260	denovo27225	denovo34341	denovo40471	denovo47139	denovo53387	denovo61985	denovo69429	denovo76099	denovo9137	denovo77863	denovo43800	
denovo27489	denovo81918	denovo7493	denovo14288	denovo21276	denovo27332	denovo34394	denovo40543	denovo47155	denovo53565	denovo62052	denovo69470	denovo76155	denovo11302	denovo80002	denovo44372	
denovo27618	denovo113	denovo7504	denovo14594	denovo21322	denovo27371	denovo34409	denovo40610	denovo47178	denovo53669	denovo62261	denovo69480	denovo76399	denovo11335	denovo80329	denovo44632	
denovo29320	denovo127	denovo8480	denovo14709	denovo21352	denovo27508	denovo34413	denovo40659	denovo47268	denovo53721	denovo62397	denovo69514	denovo76438	denovo13215	denovo80698	denovo46051	
denovo32541	denovo361	denovo9279	denovo15122	denovo21402	denovo27598	denovo34504	denovo40681	denovo47457	denovo53933	denovo62476	denovo69657	denovo76478	denovo15549	denovo69560	denovo47944	
denovo33377	denovo486	denovo9303	denovo15131	denovo21473	denovo27615	denovo34589	denovo40696	denovo47534	denovo53945	denovo62763	denovo69928	denovo76629	denovo15641	denovo56208	denovo51988	
denovo35524	denovo492	denovo9310	denovo15352	denovo21724	denovo28305	denovo34650	denovo40742	denovo47635	denovo54267	denovo62834	denovo69950	denovo76656	denovo16903	denovo804	denovo54500	
denovo37423	denovo547	denovo9359	denovo15449	denovo21806	denovo28362	denovo34684	denovo41649	denovo47674	denovo54379	denovo62898	denovo69986	denovo76698	denovo20121	denovo4940	denovo54989	
denovo37620	denovo656	denovo9630	denovo15564	denovo22475	denovo28429	denovo34695	denovo41873	denovo48099	denovo54440	denovo62942	denovo70307	denovo76858	denovo20575	denovo5498	denovo55998	
denovo38222	denovo762	denovo9688	denovo15640	denovo22527	denovo28447	denovo34881	denovo42098	denovo48356	denovo54889	denovo62968	denovo70743	denovo76926	denovo20924	denovo6152	denovo58453	
denovo39822	denovo787	denovo10010	denovo15868	denovo22682	denovo28576	denovo34908	denovo42345	denovo48358	denovo55125	denovo62975	denovo70780	denovo76994	denovo21314	denovo6830	denovo58858	
denovo40807	denovo875	denovo10471	denovo15882	denovo22798	denovo28705	denovo35050	denovo42519	denovo48511	denovo55375	denovo63178	denovo71086	denovo77545	denovo21464	denovo6923	denovo59597	
denovo41172	denovo1454	denovo10481	denovo16058	denovo23026	denovo29093	denovo35116	denovo42554	denovo48619	denovo55613	denovo63436	denovo71248	denovo77655	denovo22334	denovo8498	denovo60071	
denovo42215	denovo1564	denovo10555	denovo16096	denovo23267	denovo29314	denovo35474	denovo43009	denovo48789	denovo55678	denovo63573	denovo71357	denovo77792	denovo22630	denovo9935	denovo60547	
denovo43144	denovo1899	denovo10890	denovo16568	denovo23280	denovo29593	denovo35705	denovo43409	denovo48958	denovo56175	denovo63747	denovo71580	denovo78165	denovo22807	denovo10078	denovo61416	
denovo44905	denovo1946	denovo10953	denovo16605	denovo23281	denovo30000	denovo35805	denovo43453	denovo49046	denovo56210	denovo63811	denovo71626	denovo78205	denovo22998	denovo11434	denovo61697	
denovo46081	denovo2006	denovo10959	denovo16624	denovo23533	denovo30080	denovo36082	denovo43530	denovo49262	denovo56589	denovo63852	denovo71656	denovo78360	denovo26275	denovo12005	denovo61962	
denovo47045	denovo2011	denovo10978	denovo16803	denovo23740	denovo30203	denovo36084	denovo43693	denovo49282	denovo56602	denovo64440	denovo71936	denovo78494	denovo27630	denovo12333	denovo63898	
denovo47491	denovo2163	denovo11033	denovo17463	denovo23833	denovo30349	denovo36346	denovo44173	denovo49428	denovo56865	denovo64662	denovo71978	denovo78511	denovo30949	denovo14235	denovo66671	
denovo47654	denovo2212	denovo11137	denovo17506	denovo24112	denovo30783	denovo36684	denovo44179	denovo49460	denovo56990	denovo64674	denovo72008	denovo78550	denovo31155	denovo14487	denovo68645	
denovo48998	denovo2759	denovo11433	denovo17558	denovo24130	denovo30941	denovo36821	denovo44204	denovo49496	denovo57243	denovo64726	denovo72305	denovo78561	denovo31680	denovo15326	denovo69314	
denovo50306	denovo2927	denovo11623	denovo17645	denovo24133	denovo31171	denovo37133	denovo44220	denovo49617	denovo57317	denovo64789	denovo72325	denovo78563	denovo34009	denovo15860	denovo70602	
denovo51633	denovo3084	denovo11624	denovo17791	denovo24217	denovo31216	denovo37184	denovo44229	denovo49739	denovo57557	denovo64832	denovo72376	denovo78628	denovo34824	denovo16069	denovo70857	
denovo53365	denovo3087	denovo11648	denovo17882	denovo24427	denovo31298	denovo37198	denovo44279	denovo49879	denovo58004	denovo65380	denovo72380	denovo78751	denovo38828	denovo17316	denovo71470	
denovo53712	denovo3112	denovo11651	denovo18115	denovo24509	denovo31452	denovo37213	denovo44349	denovo50487	denovo58445	denovo65430	denovo72751	denovo78812	denovo40718	denovo17588	denovo72175	
denovo54194	denovo3222	denovo12047	denovo18146	denovo24610	denovo31679	denovo37363	denovo44505	denovo50546	denovo58448	denovo65649	denovo72765	denovo78942	denovo43945	denovo18906	denovo72516	
denovo54533	denovo3256	denovo12050	denovo18175	denovo24629	denovo31714	denovo37395	denovo44525	denovo50602	denovo58449	denovo65693	denovo73000	denovo78982	denovo44604	denovo22827	denovo72942	
denovo56116	denovo3291	denovo12142	denovo18240	denovo24653	denovo31903	denovo37737	denovo44652	denovo50613	denovo58500	denovo65721	denovo73097	denovo79019	denovo48226	denovo23462	denovo73157	

k__Bacteria.p__Firmicutes.c__Cl ostridia.o__Clostridiales.f__Pept ococcaceae
denovo75118

k__Bacteria.p__Firmicutes.c__Clostridia.o__Clostridiales.f__Ruminococcaceae.g__Butyricicoccus
denovo4477
denovo8597
denovo11917
denovo21884
denovo22988
denovo52406
denovo57662
denovo73487

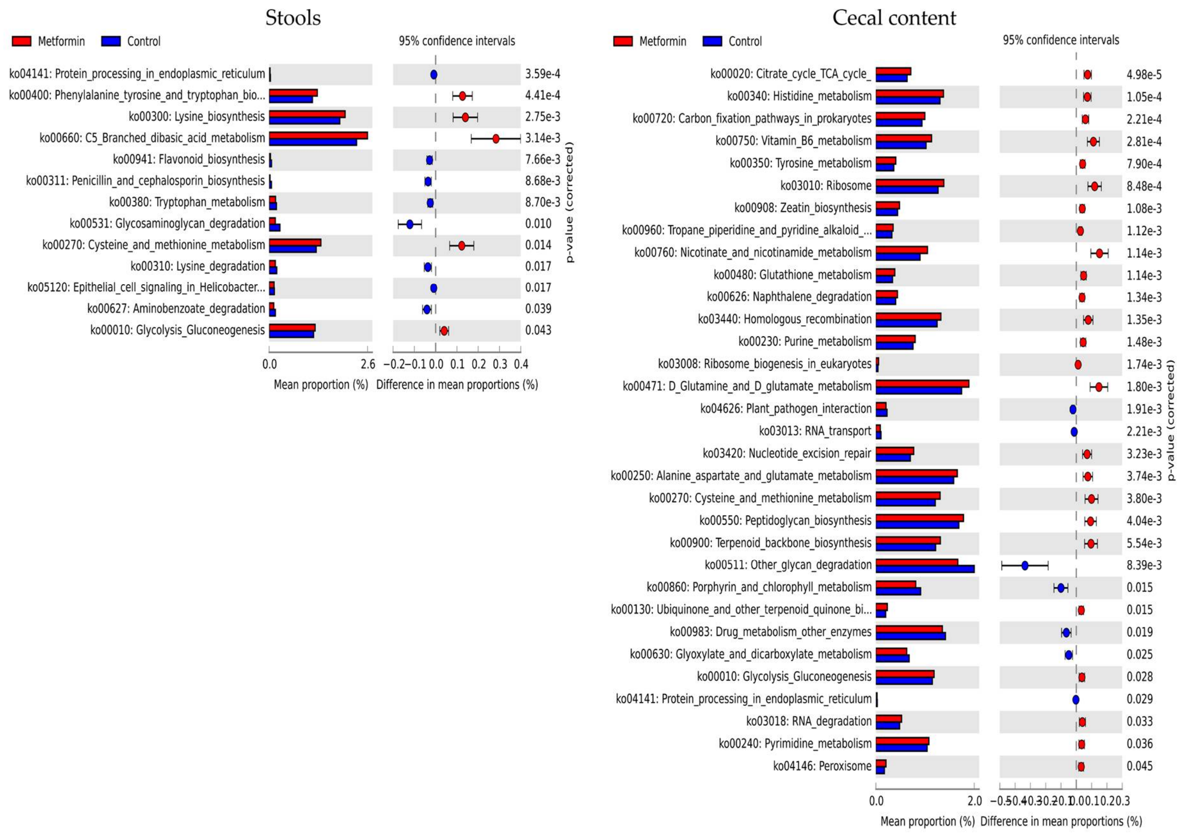

Figure S3. Differentially enriched Kyoto Encyclopedia of Genes and Genomes (KEGG) pathways (i.e., relative abundance > 0.001%) in cecal and fecal microbiota (p < 0.05).

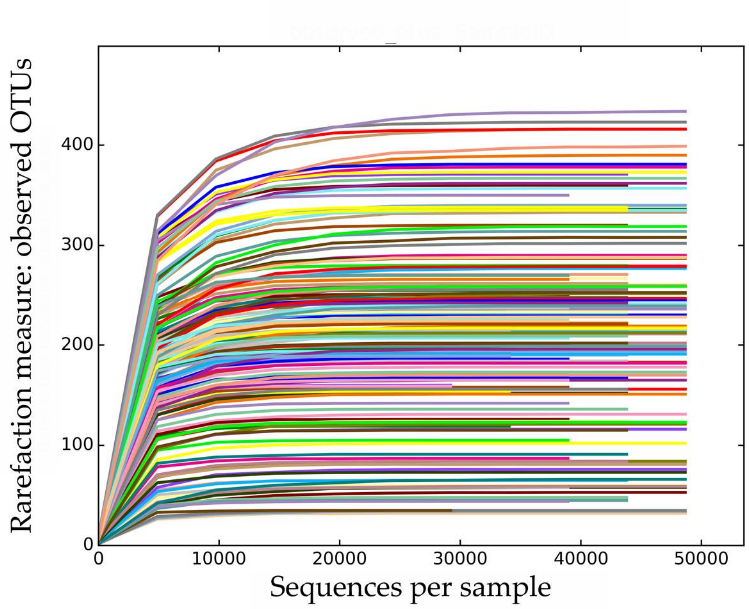

Figure S4. Rarefaction curves showing the number of observed OTU as a function of the number of sequences per samples. All samples (153) analyzed in this study were presented.

Table S4. Number of reads obtained in each sample after preprocessing.

Samples	Number of reads	Samples	Number of reads	Samples	Number of reads	Samples	Number of reads
1	79645	40	64949	79	63410	118	78084
2	55989	41	64579	80	52032	119	102842
3	92015	42	64265	81	57533	120	86872
4	58957	43	57574	82	59439	121	95966
5	77663	44	66610	83	54356	122	78709
6	70640	45	57122	84	50410	123	88562
7	87986	46	83783	85	45497	124	89355
8	66420	47	64824	86	62147	125	86426
9	84913	48	60005	87	64362	126	81709
10	95964	49	63235	88	51869	127	105105
11	91683	50	62651	89	52023	128	95330
12	72678	51	64494	90	59943	129	123933
13	87168	52	69597	91	41804	130	84681
14	66789	53	59757	92	62311	131	89451
15	79866	54	70762	93	54698	132	82373
16	65482	55	68541	94	42045	133	126809
17	66102	56	61862	95	61350	134	110943
18	62044	57	67608	96	44858	135	85467
19	57239	58	55190	97	32606	136	82502
20	87881	59	62301	98	53454	137	85507
21	66821	60	71317	99	45075	138	109549
22	86593	61	53220	100	44837	139	95059
23	104498	62	59081	101	59142	140	45628
24	78768	63	51903	102	51582	141	55684
25	74352	64	69273	103	50626	142	63501
26	53154	65	57854	104	41510	143	60142
27	70604	66	67666	105	52298	144	59139
28	71264	67	60616	106	45921	145	62810
29	71625	68	52588	107	48307	146	63360
30	80648	69	56615	108	51070	147	60401
31	65641	70	60102	109	61448	148	70067
32	65373	71	65017	110	53567	149	63861
33	55860	72	51024	111	58184	150	54678
34	64601	73	58001	112	61833	151	50451
35	74087	74	61585	113	81673	152	56174
36	82083	75	49701	114	77242	153	55721
37	41921	76	54286	115	74677		
38	71215	77	62250	116	66769		
39	63023	78	59153	117	88884		

Supplementary material S1

16SrRNA gene sequencing

DNA quantified using a SYBR Green assay (SYBR Green I, Sigma-Aldrich, Missouri, USA). For all samples, DNAg concentrations were > 2ng/µL and sufficient for analysis. Primers (343F et 803R) targeting the V3–V4 region of the 16S rRNA gene and a Metabiote

kit were used to prepare the amplicon library. Illumina MiSeq paired-end 2 × 250 bp (Illumina, San Diego, CA, USA) sequencing of the corresponding products was performed. On average, 66,333 full-length V3-V4 region of 16S rDNA sequences assembled at 97% nucleic identity were obtained. These full-length V3-V4 region of 16S rDNA sequences were qualitatively and quantitatively sufficient to allow affiliation, to obtain the taxonomic profiles of bacterial populations identified within the samples. The data were processed according to the QIIME pipeline. The pre-processing parameters included the removal of PCR primers and poor quality readings (score below Q30). Then, the minimum overlap area of 30 bases to perform reassociation was search, and finally 97% nucleic identity over the entire overlap area were assembled. Chimeric sequences were detected and eliminated among the full-length 16S rDNA sequences using an internal method based on application of the Usearch 6.1 software. Next, a clustering step was performed on group similar sequences using a defined nucleic identity threshold (97% identity for affiliation at the genus level on the targeted region of the 16S rRNA gene) using the Uclust v1.2.22q program. The number of sequences after preprocessing were available in Table S4. An open reference operational taxonomical unit (OTU) creation process and full-length binding method were used to create groups of sequences. The most abundant sequence of each OTU was then considered, this reference sequence was taxonomically compared to the Greengenes ver. 13_8 reference database (www.greengenes.gov) using the RDP v2.2 classifier. Rarefaction curves of observed OTUs indicating enough sequencing depth (Figure S4). Closed-reference OTUs were also created to allow the use of the PICRUSt software.
